# Mitochondria and Alzheimer’s Disease: the Role of Mitochondrial Genetic Variation

**DOI:** 10.1007/s40142-018-0132-2

**Published:** 2018-03-01

**Authors:** Perry G. Ridge, John S. K. Kauwe

**Affiliations:** Department of Biology, Brigham Young University, 4102 LSB, Provo, UT 84602 USA

**Keywords:** Alzheimer’s disease, Mitochondrial genetics, Mitochondrial cascade hypothesis

## Abstract

**Purpose of Review:**

Alzheimer’s disease (AD) is the most common form of dementia, affects an increasing number of people worldwide, has a rapidly increasing incidence, and is fatal. In the past several years, significant progress has been made towards solving the genetic architecture of AD, but our understanding remains incomplete and has not led to treatments that either cure or slow disease. There is substantial evidence that mitochondria are involved in AD: mitochondrial functional declines in AD, mitochondrial encoded gene expression changes, mitochondria are morphologically different, and mitochondrial fusion/fission are modified. While a majority of mitochondrial proteins are nuclear encoded and could lead to malfunction in mitochondria, the mitochondrial genome encodes numerous proteins important for the electron transport chain, which if damaged could possibly lead to mitochondrial changes observed in AD. Here, we review publications that describe a relationship between the mitochondrial genome and AD and make suggestions for analysis approaches and data acquisition, from existing datasets, to study the mitochondrial genetics of AD.

**Recent Findings:**

Numerous mitochondrial haplogroups and SNPs have been reported to influence risk for AD, but the majority of these have not been replicated, nor experimentally validated.

**Summary:**

The role of the mitochondrial genome in AD remains elusive, and several impediments exist to fully understand the relationship between the mitochondrial genome and AD. Yet, by leveraging existing datasets and implementing appropriate analysis approaches, determining the role of mitochondrial genetics in risk for AD is possible.

## Introduction

Alzheimer’s disease (AD) is the most common cause of dementia. It affects more than 20 million people worldwide, and the number of cases is expected to continue to increase [1–3]. While recent progress in understanding the genetics of the disease has been encouraging [4–10], there remain no effective strategies for the prevention or cure of AD.

In 2010, Swerdlow et al. [11] proposed the mitochondrial cascade hypothesis of AD. Briefly, an individual’s genetics determine baseline mitochondrial function and how mitochondria change as a person ages and is exposed to various environmental insults. Declining mitochondrial function then results in AD-specific pathology. This hypothesis receives support from several lines of evidence that suggest an important role of mitochondrial dysfunction in AD. First, mitochondria fundamentally change in a number of ways in AD. The rate of metabolism decreases [12], mitochondrial fusion and fission are disrupted [13], and mitochondrial concentration (i.e., the ratio of mitochondrial genomes to nuclear genomes) decreases in cerebrospinal fluid [14, 15]. In addition, morphological changes in the mitochondria, such as abnormal sizes and shapes (including both enlarged, very small, and elongated mitochondria [16–18], and reduced numbers of cristae [16]), are observed [11, 19], and enzymes of the electron transport chain encoded in the mitochondrial genome are altered and expression changes [12, 20, 21]. Amyloid plaques are known to aggregate in mitochondria [22, 23] and many of the changes noted above take place near amyloid plaques [24]. Finally, efficient mitochondrial proteostasis helps offset the effects of aggregating amyloid-β [25].

Patterns of inherited risk for AD also suggest a role for the maternally inherited mitochondria. Individuals with a maternal family history of AD have a higher risk of AD compared to individuals with a paternal family history of AD (three to nine times higher) [26, 27], or no family history, score lower on cognitive tests [28], have a lower age of onset [26, 29], and have more pronounced brain abnormalities consistent with AD (cerebral metabolic [30], higher Aβ burden [31], reduction in gray matter volume [32, 33], and increased global PiB uptake PiB-PET [34]). It has also been demonstrated that some of these brain abnormalities are associated with mitochondrial haplotypes [35]. Maternal-specific changes in risk and related AD phenotypes could be driven by X-linked AD risk, maternal-specific genetic imprinting, and mitochondrial genetic effects. To our knowledge, there is no published work implicating maternal imprinting or the X chromosome in AD risk. Several mitochondrial haplogroups/SNPs (Table 1) have been reported to correlate with AD [3].

**Table 1:** Summary of mitochondrial haplogroups, clusters, and SNPs that affect risk for AD

Haplogroup/SNP/cluster	Year	Effect	Ethnicity	Dataset size (case/control)	Dataset type
Mitochondrial haplogroups H and V					
HV [36]	2009	Risk	Eastern European	222/252	12 SNPs
HV [37]	2011	Risk	Eastern European	422/318	Positions 16024–576 genotyped (whole control region) and 11 additional SNPs
H [38]	2007	Risk	Iranian	30/100	Positions 16024–16383 (HVS-I region) sequenced
H [37]	2011	Risk	Eastern European	422/318	Positions 16024–576 (whole control region), 11 additional SNPs
H [39]	2011	Risk	Spanish	300/250 and 200/250^a^	7 SNPs
H5/H5A [40]	2010	Risk	Italian	936/776	Positions 16024–576 (whole control region) sequenced
H6A1A/H6A1B [41]	2012	Protective	Caucasian	101/632	Whole mitochondrial genomes
Mitochondrial haplogroups U and K					
UK [42]	2010	Risk	Caucasian	170/188	138 SNPs
UK, males only [37]	2011	Protective	Eastern European	422/318	Positions 16024–576 (whole control region), 11 additional SNPs
U, males only [43]	2004	Risk	Unlisted (likely Caucasian)	989/328	10 SNPs
U [38]	2007	Risk	Iranian	30/100	Positions 16024–16383 (HVS-I region) sequenced
U [44]	2001	Protective	Italian	213/389	10 restriction sites
U, females only [43]	2004	Protective	Unlisted (likely Caucasian)	989/328	10 SNPs
U5B1 or U5B1B2 [45]	2013	Risk	Caucasian	154/175	138 SNPs
U5A1 [37]	2011	Protective	Eastern European	422/318	Positions 16024–576 (whole control region), 11 additional SNPs
K [44]	2001	Protective	Italian	213/389	10 restriction sites
K [37]	2011	Protective	Eastern European	422/318	Positions 16024–576 (whole control region), 11 additional SNPs
K1A1B or K1A1B2A1 [45]	2013	Risk	Caucasian	154/175	138 SNPs
K1A [37]	2011	Protective	Eastern European	422/318	Positions 16024–576 (whole control region), 11 additional SNPs
Mitochondrial haplogroups J and T					
JT [37]	2011	Protective	Eastern European	422/318	Positions 16024–576 (whole control region), 11 additional SNPs
J [46]	2012	Risk	African American and Caucasian	3075^b^	138 SNPs^b^
J1B1 [37]	2011	Protective	Eastern European	422/318	Positions 16024–576 (whole control region), 11 additional SNPs
T, females only [37]	2011	Protective	Eastern European	422/318	Positions 16024–576 (whole control region), 11 additional SNPs
T [46]	2012	Risk	African American and Caucasian	3075^b^	138 SNPs^b^
Other mitochondrial haplogroups					
L1 [47]	2014	Risk	African American	902/187^c^	138 SNPs
B4C1 [48]	2009	Risk	Japanese	96/96	Whole mitochondrial genomes
B5 [49]	2015	Risk	Han Chinese	341/435 and 371/470^a^	Control region sequenced and 2 additional SNPs
G2A [48]	2009	Risk	Japanese	96/96	Whole mitochondrial genomes
N9B1 [48]	2009	Risk	Japanese	96/96	Whole mitochondrial genomes
Other mitochondrial DNA variation					
Increased mtDNA rearrangements^d^ [50]	2016	N/A	Unlisted	13/12	Whole mitochondrial genomes
mtDNA controls mitochondrial copy number^e^ [51]	2014	N/A	Caucasian	101/632	Whole mitochondrial genomes
mtDNA deletions^f^ [52]	2012	Risk	Unlisted	10/6	RT-PCR of mtDN4 and mtDN1
m.154244A>G [46]	2012	Risk	African American and Caucasian	3075^b^	138 SNPs^b^
m.14178T>C [46]	2012	Risk	African American and Caucasian	3075^b^	138 SNPs^b^

^a^The first set of numbers refers to the discovery dataset, the second to the replication dataset

^b^All participants were cognitively healthy at the start of the study. The researchers followed study participants for about 10 years, and measured cognition at years 2, 4, and 7 using the Modified Mini-Mental State Examination (3MS) and the Digit Symbol Substitution Test (DSST). One hundred thirty-eight SNPs were used for association analyses and whole mitochondrial genome sequences were acquired for follow-up from 138 study participants

^c^Study focused on dementia/no dementia, as opposed to AD/controls

^d^The authors identified a higher relative proportion of mtDNA rearrangements in the brains of AD patients

^e^Mitochondrial copy number is reduced in AD. The authors identified two mitochondrial haplogroups, U5A1 and T2, with significantly higher mitochondrial copy numbers compared to all other haplogroups. The authors further identified a possibly functional variant, m.9667A>G, which could lead to increased expression of TFAM. Increased TFAM has a protective effect in AD. The identified variant could plausibly protect against AD [53]

^f^The authors observed an increase in mtDNA deletions, which in turn created a cytochrome C oxidase deficiency in hippocampal cells of AD patients

This mitochondrial impact on AD risk could be influenced by several factors. Here, we review what is known about the association of variation in the mitochondrial genome with risk for AD and comment on methods for increasing the available data for answering this important question.

## Mitochondrial Genetics

Each mitochondrion possesses one or more copies of its own circular genome. The human reference mitochondrial genome (mtDNA) is 16,569 base pairs long and encodes 37 total genes: two ribosomal RNAs, 22 tRNAs, and 13 protein coding genes [54]. Each protein coding gene encodes a component of the electron transport chain, seven from complex 1 (NADH dehydrogenase subunits), three from complex 4 (cytochrome c oxidase subunits), two from complex 5 (ATP synthase subunits), and 1 from complex 3 (cytochrome b). The majority of mitochondrial proteins, however, are nuclear encoded [55]. While several nuclear-encoded candidate genes have been investigated, the largest and best powered studies of the nuclear genome have failed to identify associations between these genes and risk for AD [8]. Germline variation in the mtDNA is responsible for numerous neurological diseases (e.g., Leber hereditary optic neuropathy [56], Leigh syndrome [57], neuropathy, ataxia, and retinitis pigmentosa syndrome [58], myoclonic epilepsy with ragged red fibers [59], mitochondrial encephalomyopathy, lactic acidosis, and stroke-like episodes [60], etc.) or affects risk (e.g., Parkinson’s disease [61]) and are believed to have a pivotal role in aging [62].

**Fig. 1:**
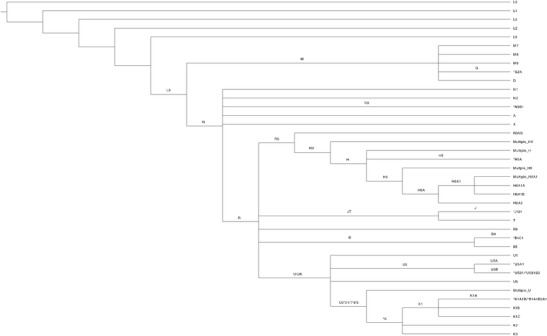
Mitochondrial network. The network includes all major mitochondrial haplogroups and subgroups described in the manuscript. The root of the tree starts with the hypothetical Mitochondrial Eve. Edges are labeled if they give rise to two or more named haplogroups. For example, L3 gives rise to haplogroups M and N. Asterisk indicates that not all branching required to traverse the network to the group is shown. However, in all cases, sufficient branching is included to show the relative relationships between all major haplogroups and subgroups described in the manuscript. Lastly, in several locations, a node label is proceeded by “Multiple_,” which indicates that multiple groups have been compressed into a single label. For example, there are numerous H6 subgroups (e.g., H6A, H6B, etc.). However, for the purposes of this manuscript, we are only interested in H6A, so other H6 subgroups are all included in the node labeled Multiple_H6. The relationships between haplogroups are based on Phylotree annotations (Build 17) [64]

Variation in the haploid mitochondrial genome is often described by established haplotype groups. In the mitochondrial phylogenetic tree, major branch points are identified as major mitochondrial haplogroups and are represented by a single letter (e.g., H, V, L, etc.), while mitochondrial subgroups are further defined by additional numbers/letters (e.g., H51A1, L3, etc.) [63]. Clusters are groups of closely related major haplogroups (e.g., HV, UK, etc.). Each mitochondrial haplogroup and subgroup are defined by a specific set of one, or several, mitochondrial SNPs, and each group and haplogroup consists of a number of distinct, but closely related from an evolutionary standpoint, mitochondrial haplotypes [63]. Mitochondrial haplogroups and subgroups identified as associated with AD could be driven by one or multiple of the SNPs defining the group when using whole mitochondrial genome sequence data. When using limited SNP data, associations could be driven by one or multiple of the SNPs defining the group, or by subgroups that are tagged by those SNPs. Here, we review the mitochondrial clusters, haplogroups, and subgroups reported to affect risk for AD, as well as specific SNPs when reported (summarized in Table 1 and relationships between haplogroups are illustrated in Fig. 1).

### Mitochondrial Haplogroups H and V

Mitochondrial haplogroup H and cluster HV, and subgroups, have been implicated in AD in several published reports. Haplogroup H was identified as a risk haplotype for AD [38]. Fesahat et al. used SNPs from the HVS-1 region in the d-loop region of the mtDNA to assign individuals to one of eight different mitochondrial haplogroups. Sixty-two SNPs were genotyped to be able to distinguish between major mitochondrial haplogroups. Five different SNPs were used to identify individuals with an H haplogroup. Given the relatively small number of queried SNPs, it is not possible to assign individuals to more specific mitochondrial haplogroups. Maruszak et al. [37] likewise reported that haplogroup H increases risk for AD by sequencing approximately 600 nucleotides of the control region and nine additional coding region SNPs, and comparing frequency in AD cases and controls. Neither group suggested possible causative SNPs. Coto et al. genotyped seven SNPs to assign individuals to haplogroup H [39]. While this study was small (discovery and replication datasets totaled 500 cases and 500 controls together), they did include both discovery and replication phases. Of the seven markers they analyzed, just one, m.7028C, had a significant frequency difference between cases and controls.

Cluster HV, which is a combination of haplogroups H and V, has been reported to be associated with increased risk for AD [36]. Maruszak et al. genotyped 12 mtDNA SNPs from blood (10 SNPs to determine nine different mitochondrial haplogroups, and two functional SNPs). HV was more frequent in cases than controls, even when controlling for APOE ε4 status, gender, and age of onset. Likewise, Maruszak et al. [37] reported an association between cluster HV and AD. The authors sequenced about 600 bases of the control region and genotyped nine coding region SNPs to be able to assign individuals to major mitochondrial haplogroups and related subgroups. The authors suggest that m.14766C>T may be responsible for the observed increase in risk for AD. m.14766C>T is a nonsynonymous variant located in cytochrome B, which results in a threonine to isoleucine substitution. This is the defining variant for the HV cluster.

Santoro et al. [40] sequenced the d-loop control region and genotyped additional positions in the mtDNA to assign individuals to major haplogroups, and in some cases more specific subgroups. The authors do not report specific positions outside the control region genotyped, but identified 299 mutations in AD cases outside the control region and 146 mutations in AD controls outside the control region. In total, they tested > 40 subgroups and found that individuals with subgroup H5 had increased risk for AD.

Lastly, Ridge et al. [45] identified two subgroups of haplogroup H, H6A1A and H6A1B, that are associated with reduced risk for AD. This was the first study using whole mitochondrial genomic data of which we are aware. The authors used TreeScanning [65] to group evolutionary-related subgroups together for analysis in a haplotype network. The authors considered every clade in the haplotype network with at least five samples. In the clades of interest, there were no AD cases, only controls. Moreover, the authors pinpointed three single nucleotide mutations likely responsible for the observed correlation (m.3915G>A, m.4727A>G, and m.9380G>A). All three SNPs are synonymous. The SNPs are located in electron transport genes: NADH dehydrogenase subunit 1 (ND1), NADH dehydrogenase subunit 2 (ND2), and cytochrome C oxidase subunit 3 (COX3). The use of whole genome sequence data provides additional opportunity for identifying putative functional polymorphisms.

### Mitochondrial Haplogroups U and K

Mitochondrial haplogroups U and K, and subgroups, have been reported to both increase and decrease risk for AD, with sometimes conflicting reports for a specific haplogroup or cluster. Cluster UK has been reported as both a risk [42] and protective [37] cluster. Lakatos et al. [42] used 138 mitochondrial SNPs genotyped on a SNP array, which are insufficient to definitively define all mitochondrial haplogroups, but are adequate to define large clusters such as UK, and at least some major mitochondrial haplogroups. The authors reported five different SNPs, each of which defines the UK cluster or subgroups of the cluster, as possibly driving the observed association: m.11467A>G (NADH dehydrogenase subunit 4), m.12308A>G (tRNA leucine 2), m.12372G>A (NADH dehydrogenase subunit 5), m.9698C>T (cytochrome C oxidase subunit 3), and m.16270C>T (control region). Since SNP data are incomplete, it is possible, even likely, that identified associations are tagging more specific subgroups of cluster UK. In contrast, Maruszak et al. [37] reported UK as a protective cluster in males. We previously described the data used by Maruszak et al. [37], which included sufficient genotyping to identify haplogroups of interest.

Similar to the UK cluster, haplogroup U has been reported as both a risk [38, 43] and protective [43, 44] haplogroup. Van der Walt et al. [43] genotyped 10 SNPs, sufficient to assign individuals to one of nine predominantly European mitochondrial haplogroups (H, I, J, K, T, U, V, W, X). They reported that U increases risk for AD in males, and decreases risk in females, relative to haplogroup H. Fesahat et al. [38] genotyped 62 SNPs to assign individuals to major haplogroups, and reported an increased risk of AD in individuals with the U haplogroup. Carrieri et al. [44] analyzed the relationship between APOE ε4 and mtDNA variation. They observed differences in mitochondrial haplogroup frequencies in AD controls with at least one ε4 allele compared to AD cases with at least one ε4 allele. Ten restriction sites were used to assign individuals to different mitochondrial haplogroups. Haplogroup U (and K, discussed below) appeared to neutralize the increased risk from the ε4 allele and was thus defined as protective against AD. In each of these studies, a limited number of SNPs or restriction sites were used to assign individuals to mitochondrial haplogroups, so there was insufficient data to confidently identify which haplogroup or subgroups are responsible for the observed signal.

Ridge et al. [45] analyzed the relationship between AD-specific physiological changes in the brain and variation in the mitochondrial genome. The authors sought to identify mitochondrial variation associated with 16 different imaging phenotypes and found associations for two different phenotypes (whole brain volume and percent change in temporal pole thickness), possible endophenotypes of AD. The authors used data from the Alzheimer’s Disease Neuroimaging Initiative (ADNI) [42] and identified mitochondrial variation associated with increased brain atrophy, consistent with AD. However, as genotypes were available for just 138 mitochondrial SNPs, it was sometimes not possible to assign a specific mitochondrial haplogroup to an individual. In the case of these two associations, available SNPs were only sufficient to assign individuals to two possible haplogroups. The first group consisted of individuals with mitochondrial haplogroup U5B1 or U5B1B2 and the second group K1A1B or K1A1B2A1 (discussed below). In each case, individuals in these groups experienced reductions in temporal pole thickness which would be considered evidence of increased risk for AD. In contrast to Ridge et al., Maruszak et al. [37] (dataset described above) reported U5A1 as a protective haplogroup.

The K haplogroup and subgroups have also been reported to increase and reduce risk for AD. In the same two studies just discussed, Ridge et al. [45] reported K1A1B or K1A1B2A1 as risk haplogroups, while Maruszak et al. [37] reported K1A as protective. Maruszak et al. [37] suggests m.497T as a possible functional variant driving the observed protection. m.497T is a control region variant, and it is not clear how it might affect mitochondrial function. Finally, Carrieri et al. [44] and Maruszak et al. [37] both reported that the K haplogroup reduces risk for AD, and Maruszak et al. [37] suggests that the responsible variants are m.9055G>A or m.16224C. m.9055G>A is a nonsynonymous substitution in ATP synthase F0 subunit 6 (ATP6) and results in an alanine to threonine substitution. This substitution has been frequently reported as a possible indicator of longevity [66–68] and as protective against Parkinson’s disease [69], which is consistent with a protective role against AD.

### Mitochondrial Haplotypes J and T

Mitochondrial haplogroups J and T have been implicated as risk and protective groups. The JT cluster reduces risk in females specifically [37], while individuals with J are more likely to have reduced cognition [47]. Tranah et al. [47] sequenced whole mitochondrial genomes and in parallel genotyped 138 mitochondrial SNPs using a SNP array for each study participant. Individuals with haplogroup J were more likely to experience declines in 3MS scores. Although the authors sequenced whole mitochondrial genomes, association analyses were only performed on major haplogroups using SNP data from the arrays, so reported results might be tagging subgroups. In contrast, J1B1 appears to have a protective role [37]. J1B1 is defined by m.497T, which is possibly responsible for the observed effect.

The T haplogroup has been reported to have conflicting roles [37, 47]. In each case, only a sufficient number of mitochondrial SNPs were used to assign individuals to major mitochondrial haplogroups and clusters, although Tranah et al. [47] sequenced the entire mitochondrial genome (they only used 138 SNPs for this particular analysis). It is possible that both are true associations and are tagging different subgroups of T. The association reported by Maruszak et al. [37] is specific to females only.

### Other Mitochondrial Haplogroups

Subgroups of B, G, N, and L have been reported to have a role in AD. B4C1 and B5 are risk groups [48, 49]. B4C1 was identified by first selecting Japanese AD patients, identifying the mitochondrial haplogroups possessed by any AD cases, then using a modified neural network (radial basis function) to identify haplogroups of interest. Specific haplogroups were identified by examining differences between AD cases and healthy Japanese centenarians. Takasaki et al. [48], using the same approach described, also reported that G2A and N9B1 increase risk for AD.

Haplogroup B5 was identified by genotyping a sufficient number of SNPs to assign individuals to major mitochondrial haplogroups and a few subgroups and comparing haplogroup frequencies between AD cases and controls [49]. This is one of the few studies to have attempted replication, and although the association did not replicate, the effect was in the right direction, and when pooling both datasets together, the association reached significance. Finally, the authors experimentally validated their discovery. m.8584G>A defines B5 and alters mitochondrial function. In samples carrying this variant, reactive oxygen species (ROS) levels were higher, ATP levels lower, and overall mitochondrial function decreased [49].

Finally, Tranah et al. [46] used 138 mitochondrial SNPs to analyze the relationship between the mitochondrial genome and AD and dementia in a dataset of African Americans. L1 had increased risk for dementia and lower plasma Aβ levels, while individuals with L3 had overall higher Aβ levels.

## Conclusions

A thorough review of mitochondrial genetic discoveries in AD reveals that there are relatively few definitive findings. For example, numerous studies reported no relationship between mtDNA and AD [70–75], and haplogroups U and T have been reported to both increase and decrease risk for AD. Additionally, to our knowledge, with one exception [39], no identified associations have been replicated and few attempts have been made. A number of issues have likely contributed to our inability to define the relationship between the mitochondrial genome and AD including small numbers of subjects, insufficient genetic data, and technical challenges in data analysis.

Substantial resources have been committed to developing massive datasets to study the nuclear genetics of AD. For example, the Alzheimer’s Disease Sequencing Project (ADSP) [76], Alzheimer’s Disease Genetics Consortium (ADGC) [9], and the Alzheimer’s Disease Neuroimaging Initiative (ADNI) [77] each have 1000s of samples. Initially, the overwhelming majority were genotyped on SNP arrays, and in the last few years, these efforts have expanded to include exome and whole genome sequencing. In contrast, there are relatively few comparable datasets dedicated to the study of mitochondrial genetics of AD. What datasets exist are typically samples acquired and genotyped by individual researchers. This has resulted in limited numbers of samples with limited genotyping.

Interestingly, many of the large genetics consortia have actually collected data for the mitochondrial genome in addition to nuclear markers and sequences. Early SNP arrays typically included genotypes for 138 mitochondrial SNPs, while newer arrays include genotypes for 256 mitochondrial SNPs. Hudson et al. leveraged these data from several different datasets and reported no consistent evidence for association with AD [75]. Unfortunately, these SNPs are not sufficient to identify detailed haplogroup and subgroup information for all individuals. This means that unless all subgroups within a haplogroup have a consistent impact on AD, then association cannot be detected. Inconclusive findings in the large sample studied in Hudson et al. [75], for example, may be due to complete lack of association or due to heterogeneity of genetic effects within the haplogroups that were defined in the study subjects.

In this review, we focused on germline variation in the maternally inherited mitochondrial genome. However, it is also possible that somatic mutations appearing in only a few tissues and at relatively low levels compared to the maternally inherited mitochondrial genome (i.e., heteroplasmy) could play a role in AD. Moreover, although the majority of mitochondria are maternally inherited, there is precedent for low levels of paternal mitochondria to escape the selective destruction that is typical and to exist in an individual [78]. Both somatic changes and paternal inheritance of mitochondria could result in varying proportions of mutant mitochondria, which can lead to mitochondrial disease [79]. This might be especially true in the brain, which is sensitive to even subtle changes in energetics. Unfortunately, these are especially difficult to study in the context of AD for two reasons. First, if tissue-specific low levels of mutant mitochondria were responsible for disease, this would require collecting samples of brain tissue. Second, from a technical standpoint, it is difficult to identify rare mutants in a population of mixed cells. Next-generation sequencing (NGS), as described below, might help to overcome this second challenge.

Collection of NGS data provides an important opportunity to expand the study of mitochondrial genetics in AD. While sequenced exomes do not typically include mitochondrial sequence data, whole genome sequence data always includes whole mitochondrial genome sequence data. Raw data from whole genome sequencing represent an untapped resource that could be leveraged to study the mitochondrial genetics of AD. Furthermore, since NGS data rely on redundant sequencing of each base, it may be possible to analyze each read to determine the mix of mutant and wild-type alleles in mitochondria extracted from specific tissues of interest.

Technical impediments exist to using these data. As mitochondrial genomes are haploid, and the majority of algorithms for analyzing genetics data were developed specifically for diploid genomes, many existing approaches for data processing and analysis do not work well for the mitochondrial genome. We recently demonstrated that whole mitochondrial genome sequence data can be used to identify mitochondrial variants [80]. We extracted whole mitochondrial genome data from 805 whole genomes from ADNI. We outlined an approach for accurately determining mitochondrial genotypes from next-generation sequencing data of whole genomes which were > 98% accurate [80]. Both our methods and the resulting dataset are publically available, with complete mitochondrial genome sequence data, genotypes, and rich phenotypic annotations. This approach could be expanded to additional samples where whole genome sequence data have been collected, resulting in a rich resource for studying the impact of mitochondrial genetic variation on AD risk.

Approaches to data analysis may also be limiting our discoveries of associations between mitochondrial genetic variation and AD. The majority of studies used only a handful of SNPs, or focused on a few specific mitochondrial haplogroups. Even in cases where whole mitochondrial genomic data have been available, studies have been limited to a small number of markers. This is likely due to the lack of simple approaches for using more detailed genetic data for association studies. Complete sequence of the mitochondrial genome results in high-resolution haplotype data, including many singletons. This results in a large number of subgroups, each with small sample sizes, which leads to significant reductions in statistical power for association testing.

This high-resolution haplotype data can be used to estimate haplotype networks, which represent the evolutionary relationships of the haplotypes. The basic assumption of evolution-based haplotype analyses is that mutations with functional consequences are embedded within the history of the population. Advantages of these methods include the ability to pool haplotypes in meaningful ways to concentrate statistical power on evolutionarily relevant contrasts and detect phenotypically convergent but evolutionarily independent mutations. TreeScanning is a method that incorporates these concepts and a permutation-based multiple test correction to analyze both qualitative and quantitative traits [65, 81, 82]. We have previously used this approach to analyze several genes including DAPK1 [81, 83] and to study the relationship between AD and the mitochondrial genome [41, 45, 51].

In summary, there is significant evidence for the role of mitochondria in AD risk. Studies of the contribution of mitochondrial genetic variation to AD risk remain inconclusive due to small sample sizes, limited genetic data collection, and inadequate approaches to association analysis. Growing sample sizes and the more widespread use of whole genome sequence data in the study of nuclear genetic risk factors for AD can also be leveraged for the study of mitochondrial genetic variation in AD. The methods and approaches to properly leverage these new data are available and provide a positive outlook for future investigations of mitochondrial genetic contributions to AD risk.
